# bFGF Oligomeric Stability Drives Functional Performance in Human Pluripotent Stem Cells

**DOI:** 10.3390/ijms27031283

**Published:** 2026-01-27

**Authors:** Dylan E. Iannitelli, Naryeong Kim, Luladey Ayalew, Qiang Wu, Xinzheng Victor Guo, Kyle Spitler, Manasa P. Srikanth, Julien Camperi

**Affiliations:** 1 Cell Therapy Engineering and Development, Genentech, 1 DNA Way, South San Francisco, CA 94080, USAguo.victor@gene.com (X.V.G.); 2 Protein Analytical Chemistry, Genentech, 1 DNA Way, South San Francisco, CA 94080, USA

**Keywords:** human PSCs, growth factor proteins, aggregates, physicochemical/functionality characterization

## Abstract

Basic fibroblast growth factor (bFGF) and Transforming growth factor-beta (TGF-β) are key regulators of human pluripotent stem cell (hPSC) maintenance, supporting pluripotency and self-renewal. bFGF is particularly critical for sustaining the undifferentiated state and is commonly supplied through feeder-derived conditioned media. Similarly, TGF-β promotes hPSC expansion by modulating signaling pathways and contributing to a supportive stem cell niche. In this study, we investigated how the quality and variability of these growth factors influence hPSC culture performance. To address this, we developed and applied multiple physicochemical characterization methods—including size exclusion and reverse-phase chromatography—to assess growth factor purity and identify impurities across different material sources. Our findings show that certain post-translational modifications in TGF-β (e.g., oxidized variants) did not measurably affect hPSC culture. However, high temperature-dependent instability of bFGF preparations significantly altered hPSC morphology and growth. These findings underscore the need for improved quality control of growth factor components in culture media to ensure consistent hPSC maintenance, thus decreasing variability across experiments. This study highlights the value of correlating analytical physicochemical data with process performance, thereby advancing material understanding, enabling more efficient process development, and facilitating the identification of critical material attributes that affect the quality of cell therapy products.

## 1. Introduction

Human pluripotent stem cells (hPSCs), including human embryonic stem cells and induced pluripotent stem cells, are vital resources with significant potential in regenerative medicine and the development of cell therapies for pharmaceutical applications [1]. To facilitate robust hPSC culture, various methods have been developed that integrate multiple key growth components, including growth factor-enriched media, extracellular matrices, three-dimensional environmental cues, and specific strategies for promoting multicellular association [2].

Human pluripotent stem cell culture has evolved from feeder-dependent systems to chemically defined, feeder-free media to improve consistency and enable large-scale production [3]. Early defined formulations like mTeSR1 and Essential 8 successfully replaced feeder cells by providing key signaling molecules directly, allowing reliable stem cell maintenance across different cell lines while highlighting the critical importance of high-quality recombinant factors [4,5].

In these defined systems, two signaling pathways are essential for maintaining pluripotency and preventing spontaneous differentiation. The basic fibroblast growth factor 2 (bFGF/FGF2) pathway signals through FGFR–ERK/MAPK & PI3K/AKT pathways, while transforming growth factor-beta (TGF-β)/Activin/Nodal signaling operates through SMAD2/3 pathways [6,7]. Even small changes in the concentration or stability of these signaling molecules can dramatically alter cell behavior and metabolism [8].

Recombinant growth factors present significant challenges for consistent cell culture due to issues with stability and variability. bFGF is particularly problematic as it degrades rapidly at 37 °C and is prone to misfolding or aggregation, leading to reduced bioactivity [9]. Stabilization of bFGF through the addition of heparin or generating engineered variants such as FGF2-STABs and FGF2-G3 can improve functionality, but significant variation still exists between different suppliers, batches, and stabilization methods in terms of purity, stability, and biological activity [10,11,12]. This variability poses major concerns for research reproducibility and clinical manufacturing, where regulatory agencies require a risk-based evaluation of all materials used. Unfortunately, many commercial media suppliers offer limited transparency regarding sourcing and quality control practices.

To address these challenges, various chemically defined hPSC media formulations have been developed, both with and without growth factors, enabling researchers to isolate the contributions of the base medium and added growth factors. The growth-factor-free, xeno-free formulation NutriStem^®^ hPSC XF (NUT-minus) enables controlled supplementation with bFGF and TGF-β. Similarly, media like E8™ and mTeSR™ provide growth factors in separate supplements, which are then added to the basal medium at the point of use.

In addition to stabilizing bFGF, alternative approaches are being developed to overcome the issues stated above. These include hyperstable bFGF variants [13,14], engineered FGFR agonists such as VHH-based molecules [15], and bFGF-derived peptide mimetics, which can offer better stability, lower production costs, and reduced immunogenicity while maintaining biological activity [16,17,18]. Systematic evaluation of these emerging solutions remains crucial to addressing the variability and stability challenges associated with recombinant growth factors.

This study aims to address quality and variability challenges in hPSC culture systems by integrating detailed molecular analysis of growth factor preparations with functional testing in defined, feeder-free systems. To this end, various HPLC methods were developed to assess the purity of bFGF and TGF-β materials. These methods also enabled the characterization of critical material attributes, such as aggregate content and other post-transitional modifications (PTMs), which are hypothesized to influence functionality. We examined growth factor materials from diverse sources, including both wild-type and engineered bFGF, and analyzed lactate production and cellular morphology as indicators of functional performance. Notably, we found that temperature-dependent oligomeric stability of bFGF differs across vendors, with strong correlations to growth performance in hPSCs. Furthermore, we explored a synthetic peptide FGFR agonist as a promising alternative to traditional recombinant protein-based FGFs, offering notable advantages such as enhanced stability, reduced production costs, and decreased immunogenicity.

## 2. Results

### 2.1. Supplier-Dependent Physicochemical Heterogeneity in bFGF and TGF-β3

#### 2.1.1. bFGF Oligomeric State and Sequence Analysis

**Figure 1:**
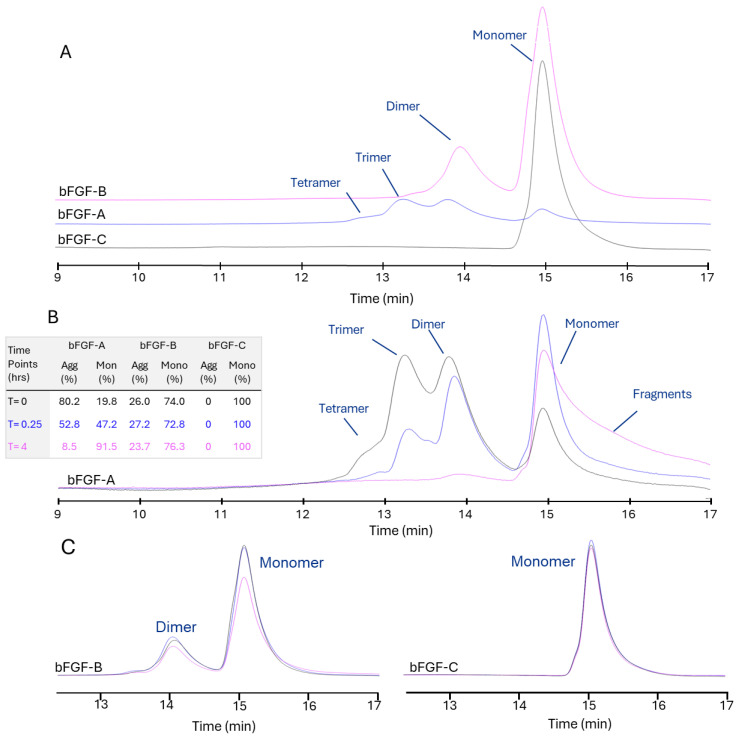
Assessment of Recombinant Human bFGF Oligomeric State from Different Vendors (A, B, C) using SEC-HPLC. (**A**) Purity profiles of bFGF materials obtained from Vendors A–C immediately after thaw at 4 °C and direct injection. Short-term stability profiles were determined by SEC-HPLC for bFGF-A (**B**) and for bFGF-B and bFGF-C (**C**). After thawing and holding aliquots at 4 °C, they were subsequently incubated at 37 °C for periods of 0, 0.25, and 4 h before analysis. Monomer (Mono., %) and aggregate (Agg., %) are quantified by peak-area integration and summarized.

Size-exclusion high-performance liquid chromatography (SE-HPLC) analysis revealed distinct source-specific differences in bFGF oligomeric distribution at baseline (Figure 1A). bFGF from Vendor C (bFGF-C) exhibited predominantly monomeric protein (99.2% monomer, 0.8% aggregate), while bFGF from Vendor B (bFGF-B) displayed a mixed population with relatively higher aggregation (74.0% monomer, 26.0% aggregate). bFGF from Vendor A (bFGF-A) demonstrated extensive aggregation with minimal monomeric content (19.8% monomer, 80.2% aggregate). Similar trends were observed across multiple batches from each vendor, with relative standard deviations of approximately 1–3% for monomer content, suggesting consistent quality between batches. Further characterization revealed that the bFGF-A aggregate forms consist of dimer, trimer, and tetramer species (Figure S1).

Time-course SEC analysis demonstrated dynamic changes in oligomeric state under assay conditions (Figure 1B). bFGF-A exhibited rapid disaggregation over 4 h, with aggregate content decreasing from 80.2% to 52.8% at 0.25 h and further to 8.5% at 4 h at 37 °C, while monomeric content correspondingly increased from 19.8% to 47.2% and 91.5%, respectively. The SEC-HPLC profile also revealed an increase in small fragments, indicating material instability and degradation at 37 °C. Conversely, bFGF-C maintained complete monomeric stability throughout the time course (100% monomer at all time points: 0, 0.25, and 4 h). bFGF-B displayed relative stability in its mixed oligomeric state, with aggregate fractions remaining consistently around 25% (26.0%, 27.2%, and 23.7% at 0, 0.25, and 4 h, respectively) and corresponding monomeric fractions of 74.0%, 72.8%, and 76.3% (Figure 1C).

To confirm these results, ELISA was used as an orthogonal method (Figure S2). The amount of bFGF quantified by ELISA correlated with the amount quantified by SEC, suggesting that the binding sites of the ELISA capture and/or detection antibodies were not accessible in the aggregate forms of bFGF. bFGF-C showed the highest concentration, corresponding to 0% aggregate forms measured by SEC. In comparison, bFGF-B had the next highest concentration, with 25% aggregate forms as measured by SEC. Furthermore, the monomer concentration of bFGF-A significantly increased with incubation at 37 °C, correlating with the rapid disaggregation of its high aggregate content. Conversely, bFGF-B and bFGF-C materials were found to be stable at 37 °C via both ELISA and SEC time-course analysis (Figure 1 and Figure S2).

#### 2.1.2. TGF-β3 Purity and Post-Translational Modification Analysis

**Figure 2:**
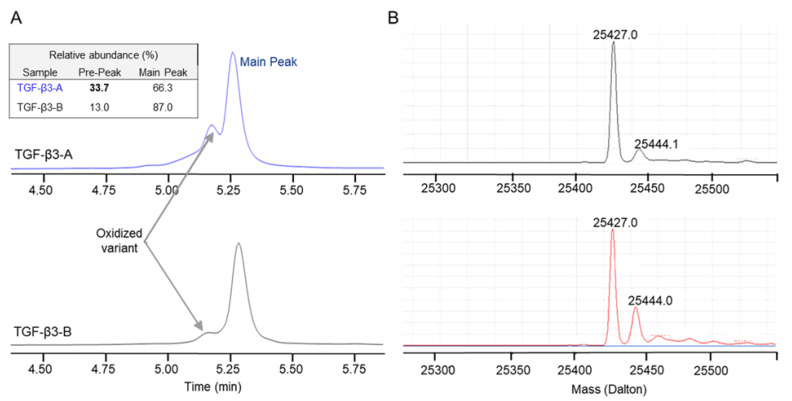
Purity assessment of TGF-β3 materials from Vendor A and Vendor B. (**A**) RP LC-UV profiles and (**B**) deconvoluted MS spectra are presented for the two materials. The pre-peak was identified as an oxidized variant, exhibiting a mass difference of +16–17 Da compared to the theoretical mass of 25,427 Da.

SEC-HPLC analysis was also employed for the characterization of TGF-β3 from Vendor A (TGF-β3-A) and TGF-β3 from Vendor B (TGF-β3-B). However, no aggregates were detected for either supplier under assay conditions. Reversed liquid chromatography-mass spectrometry (RP LC-MS) analysis revealed distinct differences in purity profiles between the two TGF-β3 materials, with TGF-β3-A visually having a higher prepeak compared to TGF-β3-B (Figure 2A). Mass spectrometry data confirmed similar primary structure, with the principal species exhibiting a mass of 25,427 Da for both materials, suggesting similar amino acid sequences (Figure 2B).

Since the sequences were similar, the observed differences likely reflected variations in post-translational modifications. Data revealed a pre-peak observed in both materials as an oxidized variant, characterized by a mass difference of +17 Da relative to the main peak (25,444 Da). Quantitative analysis revealed a supplier-dependent variation in oxidation levels. TGF-β3-B material contained approximately 13.0% oxidized variant, while TGF-β3-A contained a substantially higher amount at approximately 33.7% (Figure 2).

### 2.2. Functional Characterization of Basic Fibroblast Growth Factor Materials in Human Pluripotent Stem Cell Expansion

The physicochemical characterization of the two growth factors revealed significant source-dependent variations in oligomeric distribution and stability. To assess the impact of these variations on biological activity, functional performance was evaluated using hPSC expansion assays. These assays were designed to determine how the observed material attributes influence the capacity of the growth factors to support hPSC proliferation.

**Figure 3:**
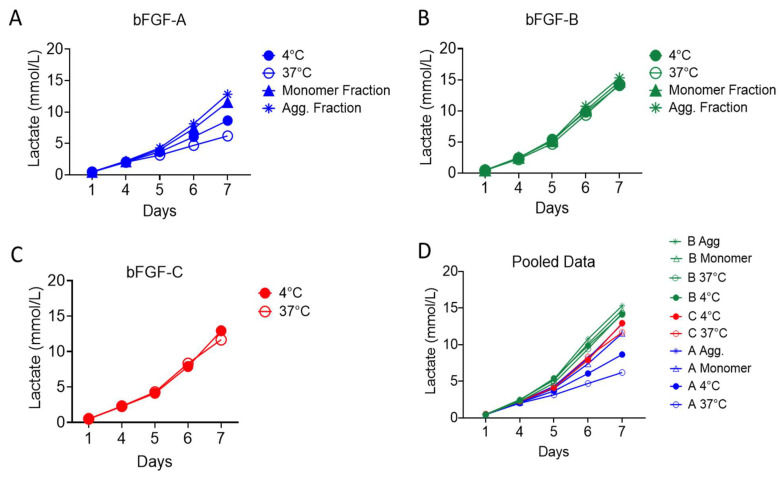
Functional characterization of basic fibroblast growth factor materials in human pluripotent stem cell expansion. (**A**–**C**) Functional performance of bFGF bulk material after thermal pretreatment (4 °C and 37 °C) and isolated fractions (Monomer and Aggregates (Agg.) from Vendor A (bFGF-A), Vendor B (bFGF-B), and Vendor C (bFGF-C). Chromatographic separations for the collection of purified monomeric and aggregated bFGF species for functional analysis are presented in Figure S3. (**D**) Pooled data comparing the hPSC expansion supported by bFGF from Vendors A, B, and C. Lactate measurements were obtained from *n* = 3 pooled wells per condition in a 6-well plate. Instrument variability, assessed across *n* = 8 replicate measurements of a single sample, yielded standard deviation values below 0.13.

The functional performance of the three bFGF materials sourced from different vendors was systematically analyzed in hPSC culture (Figure 3). hPSCs were expanded for seven days in NUT minus medium supplemented with TGFβ-3-A (constant) and each test bFGF material. hPSC growth was monitored daily by measuring lactate concentration (mmol/L) in the spent culture media.

#### 2.2.1. Impact of Thermal Pretreatment on Functional Activity

To evaluate the effects of thermal stability on biological activity, the hPSCs were fed with media that was supplemented with bFGF pre-incubated at either 4 °C or 37 °C for 4 h. Performance exhibited clear, source-specific temperature sensitivity (Figure 3A–C).

bFGF-A showed marked divergence between temperature pretreatments, with significantly reduced lactate production after 37 °C exposure. Final Day 7 lactate measurements revealed substantial differences, with 8.64 mmol/L for 4 °C pretreatment versus 6.19 mmol/L for 37 °C pretreatment (Figure 3A). This difference in functionality is consistent with the rapid disaggregation observed under elevated temperatures in physicochemical characterization.

By contrast, bFGF-B was functionally temperature-insensitive. 37 °C pretreatment did not measurably alter growth relative to 4 °C treatment, with Day 7 lactate measurements of 14.11 mmol/L and 14.27 mmol/L, respectively (Figure 3B). This thermal stability reflects the physicochemical robustness of its mixed oligomeric composition. bFGF-C (Thermostable) displayed minimal pretreatment-dependent variability, yielding Day 7 lactate concentrations of 12.93 mmol/L (4 °C) and 11.67 mmol/L (37 °C) (Figure 3C).

When comparing materials at the bulk-material level, bFGF-B material supported greater hPSC expansion than both bFGF-C and bFGF-A (B > C > A by cumulative lactate; Figure 3D). Notably, despite its near-complete monomer content and high physicochemical stability, the thermostable bFGF-C preparation exhibited lower specific functional activity than bFGF-B under the tested dosing regimen. The temperature-dependent performance of each bFGF was consistent across both laminin-521 and laminin-511 E8, demonstrating that oligomeric state and temperature stability have a greater impact on growth rate than the extracellular matrix composition (Figure S4).

#### 2.2.2. Functional Contribution of Aggregation State

To identify the active molecular species, SEC-isolated monomer and aggregate fractions from bFGF-A and bFGF-B were tested at the same nominal mass dose (Figure 3A–D). The results showed that aggregated bFGF species enable significant biological activity, essential for effective hPSC expansion (Figure 3A,B).

For bFGF-B, the purified aggregate fraction achieved the highest lactate concentration of 15.31 mmol/L by Day 7, slightly exceeding the monomer fraction (14.72 mmol/L). For bFGF-A, both purified fractions substantially outperformed bulk treatments: the aggregate fraction reached 12.82 mmol/L while the monomer fraction achieved 11.54 mmol/L, compared to bulk material performance of 8.64 mmol/L (4 °C) and 6.19 mmol/L (37 °C).

These findings demonstrate that certain aggregated bFGF species allow FGFR signaling, albeit with source-specific differences in relative activity compared to monomeric forms.

#### 2.2.3. Morphological Assessment

**Figure 4:**
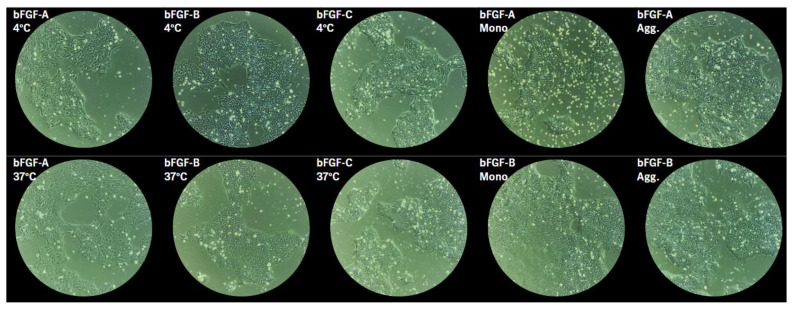
Morphological assessment of hPSCs correlates with functional growth on Day 6. Brightfield images of hPSC colonies on Day 6 after thaw, 4× magnification.

Brightfield imaging on Day 6 revealed morphology consistent with functional readouts (Figure 4 and Figure S5). For bFGF-A, cells in the 37 °C condition appear to have lower nucleus to cytoplasm ratio and less cell compaction. Cells grown with bFGF-B or bFGF-C appear to be more compacted, have higher nucleus to cytoplasm ratio, and smoother colony borders compared to cells grown with bFGF-A, regardless of incubation at 4 °C or 37 °C. There is less apparent difference by morphology between monomer versus aggregate isolated fraction for both bFGF-A and bFGF-B, though cells grown with bFGF-B monomer or aggregate appear more compacted in smooth, defined-edged colonies compared to bFGF-A fractions. In general, conditions yielding higher lactate production displayed morphology consistent with high-quality stem cell cultures [19,20]

#### 2.2.4. TGF-β3 Assessment

The significant functional dependence on bFGF source and thermal stability established bFGF as a key driver of culture variability, informing subsequent systematic evaluation of bFGF and TGF-β source contributions to overall hPSC culture performance.

**Figure 5:**
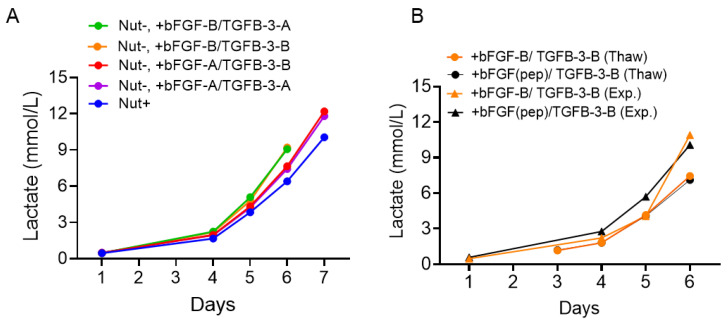
bFGF source dominates hPSC expansion performance and a peptide FGFR agonist recapitulates recombinant bFGF activity. Lactate measurements taken prior to media exchange on each day apart from weekends (Days 2 and 3), comparing different combinations of bFGF and TGF-β3 from Vendors A and B (**A**) or peptide FGFR agonist (bFGF(pep)) against bFGF. (**B**). Thaw corresponds to the growth phase directly following hPSC thawing, Exp. corresponds to the growth phase directly following passage. Lactate measurements were obtained from *n* = 3 pooled wells per condition in a 6-well plate. Instrument variability, assessed across *n* = 8 replicate measurements of a single sample, yielded standard deviation values below 0.13. Cultures were terminated when cell confluence reached ≥ 80%, consistent with standard culture practice [21,22].

After functional characterization demonstrating that the bFGF source critically influences both proliferation rate and morphology of hPSCs, we systematically mapped the interaction between bFGF and TGF-β3-A and TGF-β3-B (Figure 5). To quantify the individual effects of bFGF and TGF-β3 source on hPSC culture, we performed growth assays in NUT minus medium supplemented with bFGF and TGF-β3 sourced from either Vendor A or Vendor B, and benchmarked performance against the complete commercial medium (Nutristem plus). Growth kinetics were assessed via lactate production over seven days.

Across all tested combinations, the bFGF source exerted the greatest influence on culture performance. Conditions supplemented with bFGF-B consistently outperformed those supplemented with bFGF-A, irrespective of the TGF-β3 source (Figure 5A). By Day 6, the bFGF-B/TGF-β3-B condition achieved the highest lactate concentration (9.21 mmol/L), closely followed by the bFGF-B/TGF-β-A condition (9.07 mmol/L). In contrast, bFGF-A conditions yielded lower lactate levels (bFGF-A/TGF-β3-B: 7.65 mmol/L; bFGF-A/TGF-β3-B: 7.43 mmol/L), comparable to or only marginally higher than the NUT plus control (6.40 mmol/L by Day 6). Cell morphology was consistent with increased lactate production: hPSCs cultured with bFGF-B were more compact, exhibiting a higher nucleus-to-cytoplasm ratio regardless of the TGF-β3 source, compared with cells cultured with bFGF-A (Figure S6). The growth advantage of media supplemented with bFGF-B over the NUT plus control (Day 6 lactate concentration: NUT minus + bFGF-B/TGF-β3-B, 9.74 mmol/L; NUT plus, 5.59 mmol/L) was reproducible at larger scale (75-cm^2^ T-flasks) and across three independent lots of NUT minus base medium (Figure S7). This indicates that variability in hPSC expansion is primarily driven by the bFGF source, with bFGF-B conferring superior growth irrespective of the paired TGF-β3 source.

Given the significant impact of the bFGF source on culture performance and the known lability of rbFGF-B, we next asked whether a stabilized alternative could preserve expansion while potentially mitigating source-related variability. We benchmarked a novel FGFR agonist (bFGF(pep)) against bFGF-B under two operational contexts (“Thaw” and “Expansion”), each combined with recombinant TGF-β3, and quantified cumulative lactate levels over six days. Across all four conditions (+bFGF-B/TGF-β3-B Thaw, +bFGF(pep)/TGF-β3-B Thaw, +bFGF-B/TGF-β3-B Expansion, +bFGF(pep)/TGF-β3-B Expansion), growth kinetics were indistinguishable, converging by Day 6 to approximately 10–11 mmol/L lactate (Figure 5B). The morphology of cells cultured with bFGF(pep) versus bFGF-B/TGF-β3-B was also largely indistinguishable, with both conditions showing compacted cells with high nucleus to cytoplasm ratio and colonies with defined borders (Figure S8).

Together, these results establish bFGF source as a primary driver of hPSC expansion variability and demonstrate that a peptide FGFR agonist can match rbFGF performance across distinct workflow contexts. This alignment suggests a practical path to reduce lot-to-lot and thermal stability-related variability by substituting rbFGF with a more stable agonist, without sacrificing expansion potency.

## 3. Discussion

Defined, feeder-free media have improved standardization of hPSC culture, but they shift process sensitivity onto a small set of recombinant inputs, chiefly bFGF and TGF-β3. The material attributes and in-use stability of these growth factors drive lot-to-lot performance variability [23]. In this study, we observed vendor-specific physicochemical heterogeneity in both bFGF and TGF-β3, varying in stability, oligomeric state, and post-translational modification state. However, source-specific physicochemical variability in bFGF, not TGF-β3, drives functional performance differences in hPSCs, with temperature stability a key predictor of proliferation and morphology [9,10]. To that end, a stabilized, synthetic peptide FGFR agonist addresses the pitfalls of recombinant protein mixed oligomeric states while matching the performance of the most potent bFGF sources, providing a pathway toward lot-to-lot stability and reproducibility.

**Functional Role of bFGF Oligomeric State and Thermal Lability**. We observed striking supplier-dependent differences in bFGF oligomeric composition and primary structure, and differential oxidation in TGF-β3. In defined systems with limited “buffering” components, such material attributes readily propagate to process outputs. Prior work has documented bFGF lability and the consequences of altered stability on signaling dynamics [10,14], consistent with our finding that supplier attributes correlate with hPSC expansion kinetics. bFGF from Vendor A was found to be highly labile, displaying rapid degradation and disaggregation at 37 °C with corresponding loss in functionality. The high lability of wild-type bFGF (bFGF-A) is mainly attributed to its lack of disulfide bonds, flexible structural regions, susceptibility to chemical modifications (oxidation and deamidation), and tendency to aggregate in the absence of stabilizing interactions such as heparin [24].

In contrast, bFGF materials from Vendors B and C (mutant sequences) were much more temperature stable with corresponding improvements in cell performance. However, in isolating monomeric and aggregate fractions from bFGF-A and bFGF-B, we were surprised to find that aggregated bFGF results in higher proliferation compared to monomers. Two mechanisms can explain this finding: 1. Aggregated bFGF can engage FGFR and activate downstream signaling, raising the subsequent question of whether only dimers can bind FGFR or trimers and tetramers can as well, or 2. Aggregated bFGF acts as a stable source of monomer bFGF that releases at 37 °C to then bind FGFR, and delays the degradation to smaller, non-functional fragments. Carefully designed in vitro binding assays and three-dimensional ligand–receptor structural investigations, which are outside the scope of this study, would be required to directly address this functional phenomenon. However, previously determined crystal structures of the bFGF–FGFR complex reveal either 1:1 or 2:2:2 bFGF:FGFR:heparin stoichiometries, with no evidence for 2:1 complexes [25].

**Time–temperature handling as a hidden CPP**. Within a process-control framework, we treat growth factors as materials whose properties map to potential critical material attributes (pCMAs) such as identity/sequence, oligomeric state, chemical modifications, and potency under realistic handling, while time–temperature history during routine media preparation constitutes a key critical process parameter (CPP). This perspective aligns with modern quality systems and risk-based expectations for ancillary materials [26,27]. Our thermal pre-treatment experiments indicate that in-use functionality after realistic warm holds is the control-relevant attribute, rather than static release purity. Wildtype bFGF is unstable in culture media at 37 °C. Although heparin and engineered stabilization can reduce this instability, they cannot fully eliminate it [28]. By contrast, more robust inputs reduce process sensitivity. When potency is temperature-insensitive, the same handling window ceases to function as a CPP. Practically, we therefore define and verify allowable time–temperature envelopes per vendor/lot and incorporate use-phase functionality assays into incoming qualification.

**Activity, not generic purity, should anchor specifications**. We found that an aggregate-enriched bFGF fraction can equal or exceed the monomer fraction functionally, underscoring that % monomer or % aggregate are insufficient CMAs unless demonstrated to functionally affect biological performance. Previous studies on thermostable bFGF variants (e.g., FGF2-G3/“STABs”) similarly showed that altered biophysical signatures can preserve signaling after thermal stress, decoupling classical purity surrogates from use-phase potency [10,12]. This can then have implications on specification setting, favoring a function-first approach, potency after a defined 37 °C exposure that mimics facility Standard Operating Procedures (SOPs), over purity thresholds alone.

In a quality-by-design (QbD) paradigm, our control strategy combines (i) material qualification that includes identity/sequence (intact MS), oligomeric profile (SEC), and stress-potency (37 °C hold), (ii) standardized handling SOPs that set explicit limits on warm time and media-change cadence, and (iii) in-process indicators (early lactate trajectory, quantitative morphology) with predefined triggers for raw-material re-testing and root-cause analysis. This aligns with USP <1043> and ICH Q8–Q10 by linking material attributes to CPPs and CQAs under a documented control strategy.

We can also change the control problem by adopting inputs whose intrinsic robustness widens the operational design space. Stabilized bFGF variants (e.g., FGF2-G3/“STABs”) extend in-use half-life at 37 °C and have been used to reduce feed frequency and “weekend” interventions in pluripotent culture [10,12]. In parallel, receptor-targeted agonists, including homobivalent VHH agonists of FGFR and bFGF-derived peptide mimetics, recapitulate bFGF-like signaling with potentially improved manufacturability and stability, offering a route to lower CPP sensitivity at equivalent biological effect [29,30,31]. Our benchmarking showing that an FGFR agonist matches recombinant bFGF for expansion supports this direction.

**Context and limitations**. Historically, the move to mTeSR/E8 removed feeder-derived variability but placed greater emphasis on recombinant-factor quality and handling diligence [4,5]. Our findings extend that narrative by pinpointing which material attributes matter for hPSC expansion and by showing that use-phase potency is the appropriate target for specification and control. Two limitations remain. First, the mapping from detailed physicochemical results (e.g., specific TGF-β3 oxidation states) to potency is not fully generalizable and warrants mechanistic follow-up, and second, while lactate and morphology are useful leading indicators, standardized potency assays or broader multi-omics readouts could sharpen IPC decision-making. Addressing these gaps would enable platform-level control strategies that travel across suppliers and product variants.

In defined hPSC culture, growth-factor variability is a central control problem rather than a mere sourcing issue. A specification anchored in use-phase potency, explicit time–temperature CPP limits, and, where feasible, more robust ligands (thermostable bFGF or FGFR agonists) offer a practical route to reduce variability, expand the operational design space, and align culture performance with clinical manufacturing expectations (USP <1043>; ICH Q8–Q10).

## 4. Materials and Methods

### 4.1. bFGF and TGF-β3 Materials

Recombinant human basic fibroblast growth factor (bFGF) materials were sourced from three different vendors with comparable pricing. bFGF from Vendor A (bFGF-A) has a wild-type amino acid sequence. Conversely, bFGF from Vendor B (bFGF-B) and Vendor C (bFGF-C) have mutant sequences. These modifications, which may include deletions or amino acid replacements, are reportedly designed by the suppliers to enhance protein stability and prevent aggregation. For transforming growth factor beta 3 (TGF-β3) materials—TGF-β3-A and TGF-β3-B, procured from Vendor A and Vendor B, respectively—both possess a wild-type sequence. FGF2 Alternative Peptide (FGFR1c agonist) was purchased from PeptiGrowth Inc. (Tokyo, Japan).

### 4.2. Cell Media and Reagents

The human embryonic stem cell line HAD-C 101 (Tannenbaum, S.E. et al. 2012 Plos One) was obtained via partnership from CellCure Neurosciences Ltd., a Lineage Cell Therapeutics subsidiary founded at Hadassah Medical Center, and maintained on Laminin 521 (Biolamina, Sundbyberg, Sweden) or iMatrix-511 (Takara, San Jose, CA, USA) in Nutristem minus supplemented with TGFB3 from Vendors A or B at 2 ng/mL and bFGF from Vendors A, B, or C at 10 ng/mL, or maintained in Nutristem plus (Sartorius, Göttingen, Germany). FGFR agonist was supplemented to Nutristem minus at 3 ng/mL. Cells were single-cell passaged using TrypLE Select (Gibco, Waltham, MA, USA) and fed daily, apart from weekends.

### 4.3. Physicochemical Characterization

#### 4.3.1. SEC-UV Analysis

Growth factor proteins were analysed using a TSKgel UP-SW3000 UHP-SEC column (4.6 mm I.D. × 300 mm, 2.0 μm, 250 Å) from Tosoh (King of Prussia, PA, USA) with a mobile phase composed of 0.20 M potassium phosphate, 0.25 M potassium chloride, pH 6.2.5. The injection volume was 40 μL, and the protein concentration was 0.1 mg/mL. The separation was operated at 25 °C with a flow rate of 0.30 mL/min. A diode array detector was used for recording the UV signal at 280 nm.

Monomer and aggregate fractions were separated by SEC and then collected and subjected to buffer exchange using a 3-kDa molecular weight cutoff membrane (Millipore Sigma, St. Louis, MO, USA), followed by controlled pre-concentration. Final protein concentrations for each fraction were determined by NanoDrop (ThermoFisher, Waltham, MA, USA) absorbance at 280 nm using consistent pathlength and extinction coefficient settings. The fractions were subsequently diluted as needed to harmonize final protein concentrations between the different monomer sources, and similarly across aggregate fractions. To assess fraction purity and verify the effectiveness of the isolation procedure, each collected fraction was reinjected onto the SEC column under identical chromatographic conditions. This reinjection served as a quality control measure to confirm fraction purity. The resulting purity profiles are provided in Figure S3.

#### 4.3.2. 2D (SEC × RP) LC/MS Analysis

Aggregate fractions from ^1^D SEC column were resolved, and the corresponding peaks were diverted to the ^2^D RP-HPLC column for desalting, prior to mass spectrometry analysis. Samples were desalted and separated using a BioResolve™ RP mAb Polyphenyl (2.1 mm I.D. × 150 mm, 2.7 μm, 450 Å) column from Waters (Milford, MA, USA). The mobile phase was composed of 0.1% TFA and 0.1% TFA in water (A) and ACN (B). The gradient was configured to hold at 2% B for 10 min, ramp from 2% to 95% B over 8 min, hold at 95% B for 4 min, decrease from 95% to 2% B over 0.1 min, and finally hold at 2% for 8 min. Column temperature was maintained at 70 °C. The HPLC system was controlled by Chromeleon software (revision edition C.01.07 SR4 [version 505]) from Thermo Fisher Scientific (Waltham, MA, USA). The mass spectrometry analysis was performed on an Agilent 6230B Accurate-Mass Time of Flight (TOF) Mass Spectrometer equipped with a dual electrospray ion source (ESI). Data were acquired in an *m*/*z* range of 200–3200. Electrospray ionization was performed in positive mode and using the following source conditions: gas temperature 350 °C, gas flow 10 L/min, capillary voltage 3500 V, and fragmentor voltage 250 V. Spectra were acquired at 1.03 spectra/s with *m*/*z* range 200–3200. The MS system was controlled by Agilent MassHunter Data Acquisition software (Version B.09.00 (B9044.0)) from Agilent Technologies (Santa Clara, CA, USA). Analysis of the acquired data was performed using Agilent MassHunter Qualitative Analysis software (B.07.00).

#### 4.3.3. RP LC/MS Intact Mass Analysis

Experiments were performed on an Agilent 1290 Infinity III UHPLC system (Agilent Technologies, Santa Clara, CA, USA) equipped with a quaternary solvent delivery pump, autosampler, and UV detector. Protein analysis was performed using BioResolve™ RP mAb Polyphenyl (2.1 mm I.D. × 150 mm, 2.7 μm, 450 Å) column from Waters (Milford, MA, USA). The mobile phase was composed of 0.1% FA and 0.1% FA in water (A) and ACN (B). The gradient setup was: 1 min at 20–30%B (hold), 7 min from 30–42%B, 1 min from 42–95%B, 1 min at 95%B (hold), 1 min from 95–20%B, and 5 min at 20%B (hold). The injection volume was 5 μL, and the separation was operated at 70 °C with a flow rate of 0.30 mL/min. A diode array detector was used for recording the UV signal at 280 nm. Mass spectrometry analysis was conducted under the same conditions as those previously detailed. The LC/MS system was controlled by Agilent MassHunter Data Acquisition software (Version B.09.00 (B9044.0)) from Agilent Technologies (Santa Clara, CA, USA). Analysis of the acquired data was performed using Agilent MassHunter Qualitative Analysis software (B.07.00).

#### 4.3.4. Enzyme-Linked Immunosorbent Assay (ELISA)

Concentrations of bFGF were quantified using the Human FGF basic/FGF2 DuoSet^®^ ELISA kit (R&D Systems^®^, Minneapolis, MN, USA, Catalog # DY233). All reagents were prepared according to the manufacturer’s specifications. The primary reagent diluent was 1% BSA in PBS (pH 7.2–7.4), and the wash buffer consisted of 0.05% Tween^®^ 20 in PBS (pH 7.2–7.4). Ninety-six–well microplates were coated with 100 μL/well of mouse anti-human bFGF capture antibody and incubated overnight at room temperature. Following the coating and between all subsequent steps, plates were washed three times with 400 μL/well of wash buffer using an automated plate washer. Wells were then blocked with 300 μL/well of reagent diluent for at least 1 h at room temperature. Samples were thawed at 4 °C before the assay. A seven-point standard curve was generated using two-fold serial dilutions of recombinant human bFGF. Standards and samples were added in duplicate (100 μL/well) and incubated for 1 h at room temperature. Plates were then washed and incubated with 100 μL/well of biotinylated mouse anti-human bFGF detection antibody for 1 h at room temperature. This was followed by a 20-min incubation with 100 μL/well of streptavidin-HRP, protected from light. The reaction was developed by adding 100 μL/well of TMB substrate solution and incubating for 20 min at room temperature in the dark. The reaction was terminated with 50 μL/well of 2 N H_2_SO_4_. Optical density (OD) was measured immediately at 450 nm, with wavelength correction performed by subtracting readings taken at 570 nm. After averaging duplicate readings and subtracting the mean zero-standard OD, sample concentrations were calculated by interpolating from a four-parameter logistic (4-PL) standard curve and multiplying by the sample dilution factor.

### 4.4. Functionality Assessment

#### 4.4.1. Lactate Measurement

Spent media (1 mL) was collected into a microcentrifuge tube during each media exchange. The collected media were subsequently analyzed for lactate concentration (mmol/L) using the Cedex Bio (Roche, Indianapolis, IN, USA).

#### 4.4.2. Morphology Assessment

Stem cell cultures were observed and imaged using an Echo Revolve microscope (Echo, San Diego, CA, USA) subsequent to each media exchange, and qualitatively assessed for confluence, nucleus-cytoplasm ratio, cell separation versus compaction, spontaneous differentiation, and smooth versus jagged colony borders as morphological indicators of pluripotency.

## Data Availability

Data available on request due to restrictions e.g privacy or ethical. The data presented in this study are available on request from the corresponding author.

## Supplementary Materials

The following supporting information can be downloaded at: https://www.mdpi.com/article/10.3390/ijms27031283/s1.
