# Supplementary material for: bFGF Oligomeric Stability Drives Functional Performance in Human Pluripotent Stem Cells
Source: Int J Mol Sci. 2026 Jan 27;27(3):1283. doi: 10.3390/ijms27031283 (PMC12898039; doi:10.3390/ijms27031283)
Supplement: Supplementary file 1 [file ijms-27-01283-s001.zip › ijms-4081144-supplementary.pdf]

Supplementary Document

bFGF Oligomeric Stability Drives Functional Performance in Human Pluripotent Stem Cells

Dylan E. Iannitelli [1,†], Naryeong Kim [1,†], Luladey Ayalew [1],Qiang Wu [1], Xinzheng Victor Guo [1], Kyle Spitler [2], Manasa P. Srikanth [1,*], and Julien Camperi [1,*]

[1]Cell Therapy Engineering and Development, Genentech, 1 DNA Way, South San Francisco, CA 94080, United States.

[2]Protein Analytical Chemistry, Genentech, 1 DNA Way, South San Francisco, CA 94080, United States.

†Co-first authors.

* Correspondence: authors: srikanm1@gene.com, camperi.julien@gene.com.

Keywords: Human PSCs; growth factor proteins; aggregates; physicochemical/functionality characterization

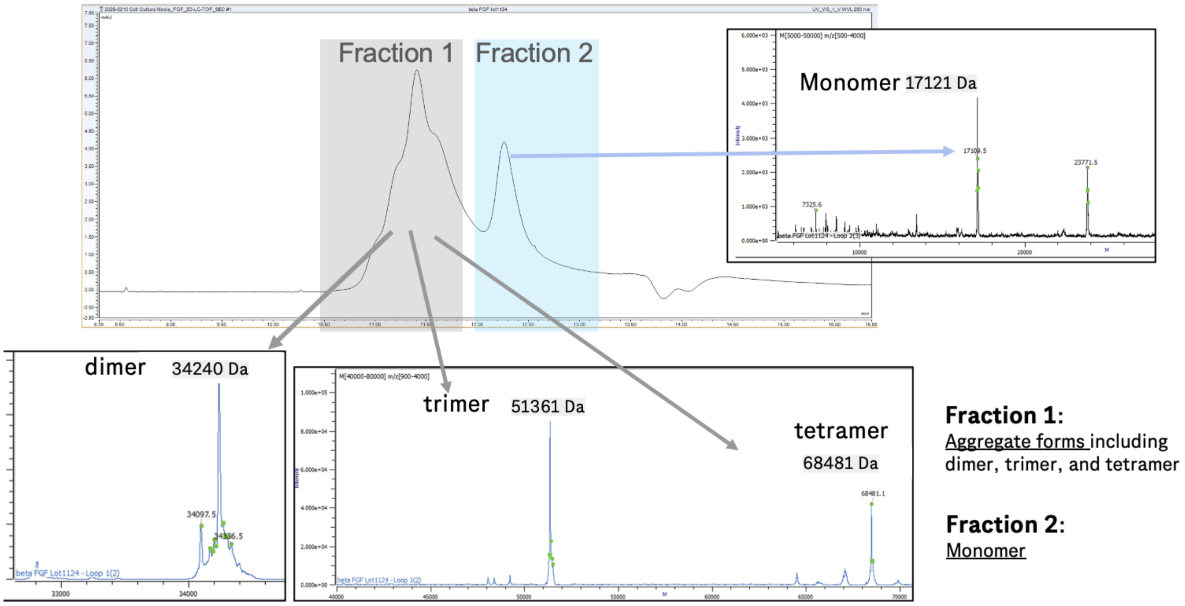

Figure S1. Physiochemical characterization of bFGF oligomeric states via 2D Size Exclusion and Reversed-Phase Chromatography Coupled with Mass Spectrometry. Deconvoluted MS spectra of each fraction are presented.

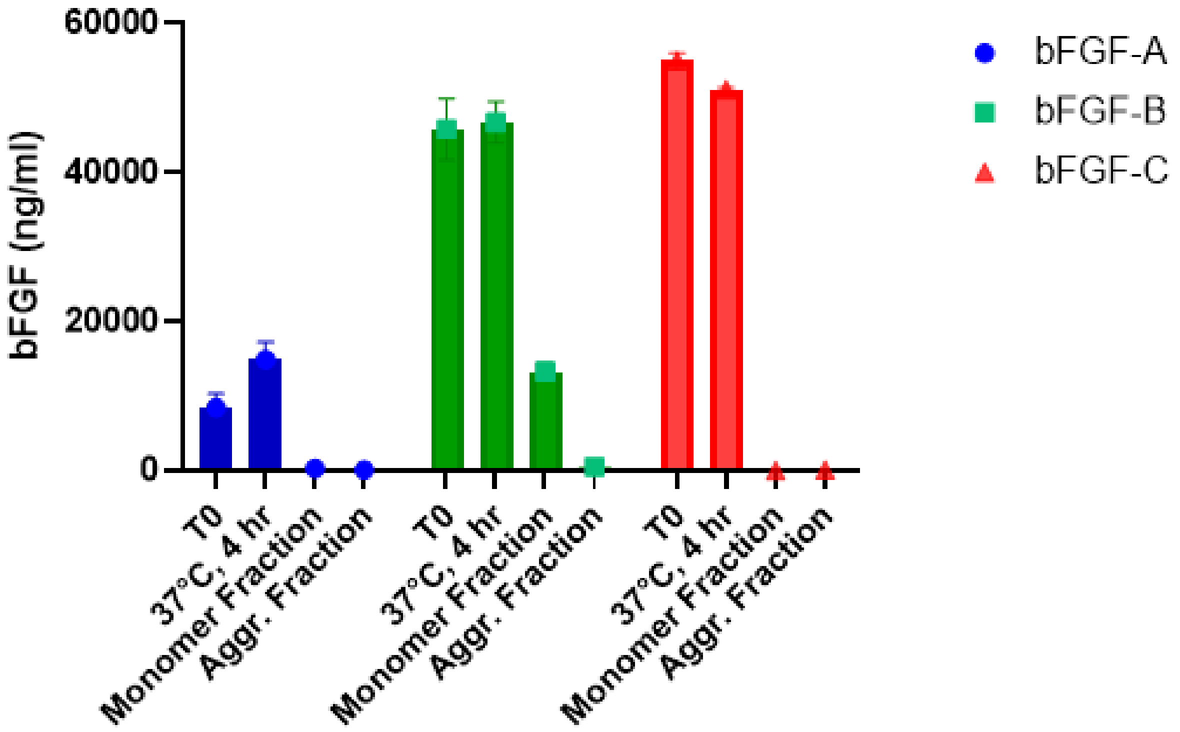

Figure S2. Enzyme-linked immunosorbent assay (ELISA) quantification of monomeric bFGF concentration correlates with thermal stability observed by SEC-HPLC.

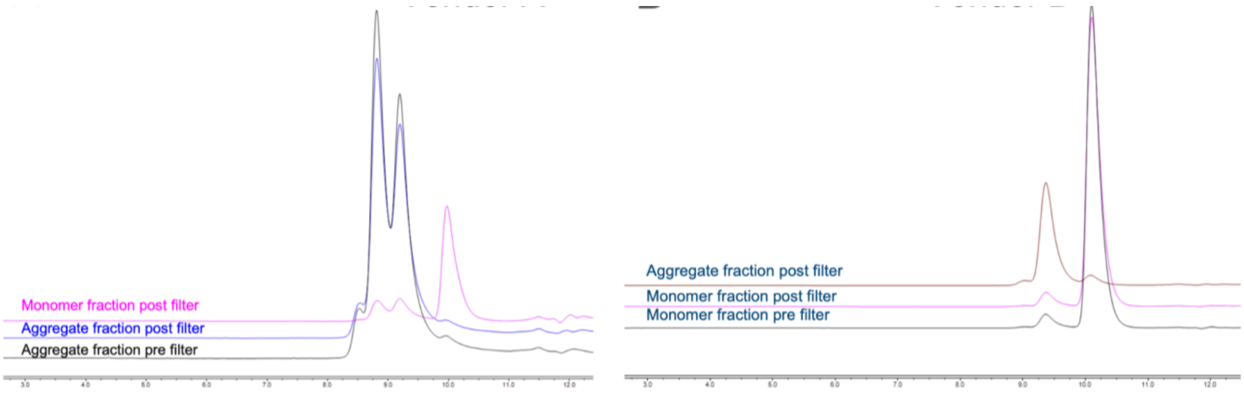

Figure S3. Chromatographic separation and collection of purified monomeric and aggregated bFGF species for bFGF-A (left panel) and bFGF-B (right panel) for functional analysis.

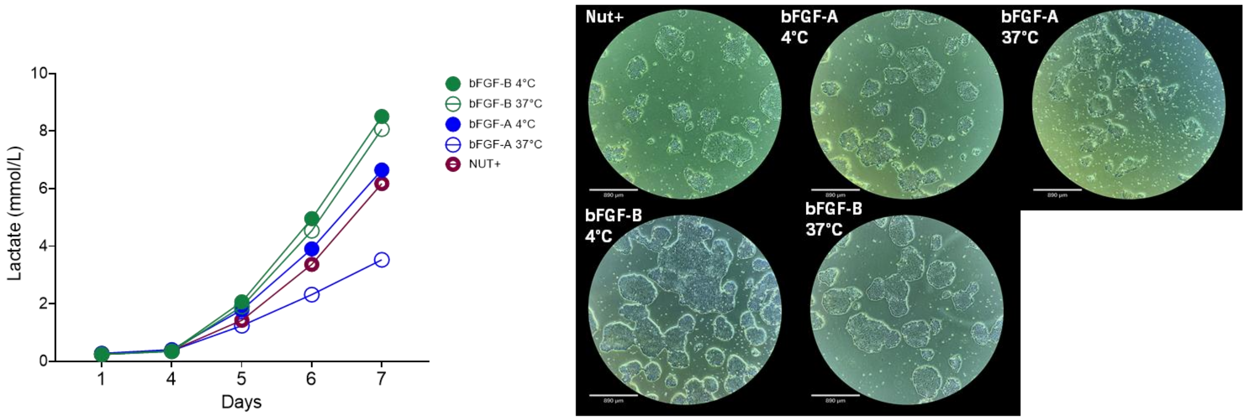

Figure S4. Functional outcome remains consistent irrespective of the substrate utilized. Lactate measurement and Morphology assessment (at day 6) for different conditions.

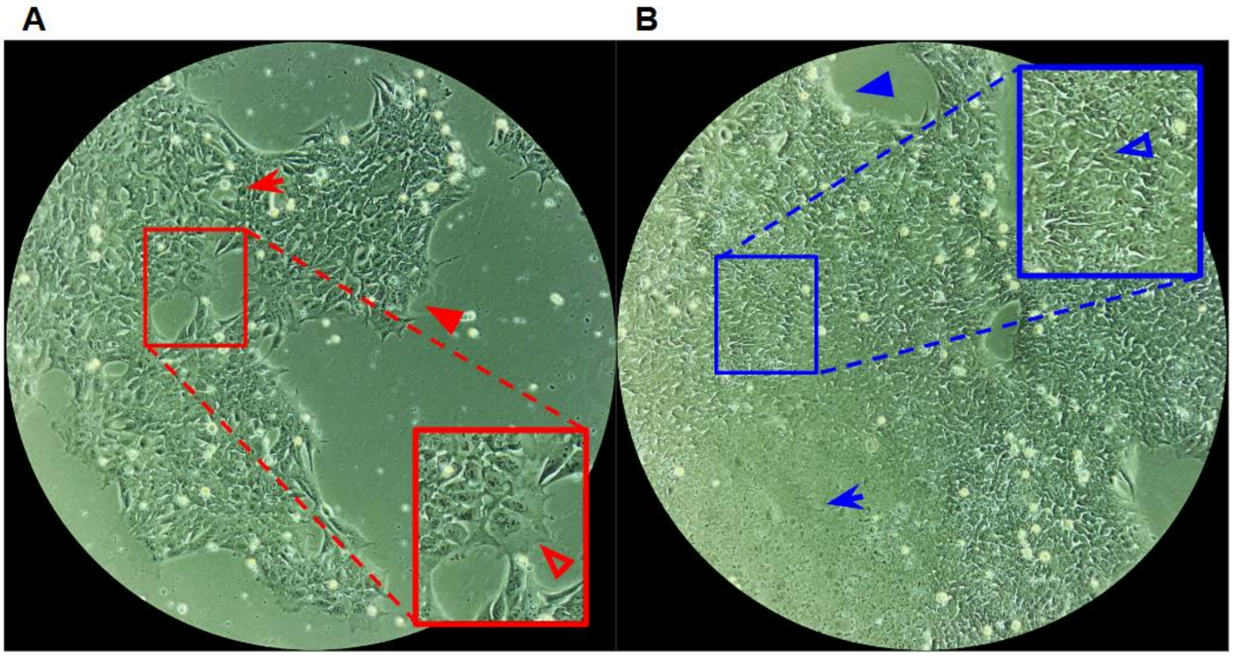

Figure S5. Morphology indicators for pluripotent stem cells. (A) Representative image of Day 6 bFGF-A 4ºC highlighting cell separation (red arrowhead), jagged colony borders (closed red triangle), and low nucleus:cytoplasm ratio (open red triangle). (B) Representative image of Day 6 bFGF-B Mono highlighting cell compaction (blue arrowhead), smooth colony borders (closed blue triangle), and high nucleus:cytoplasm ratio (open blue triangle).

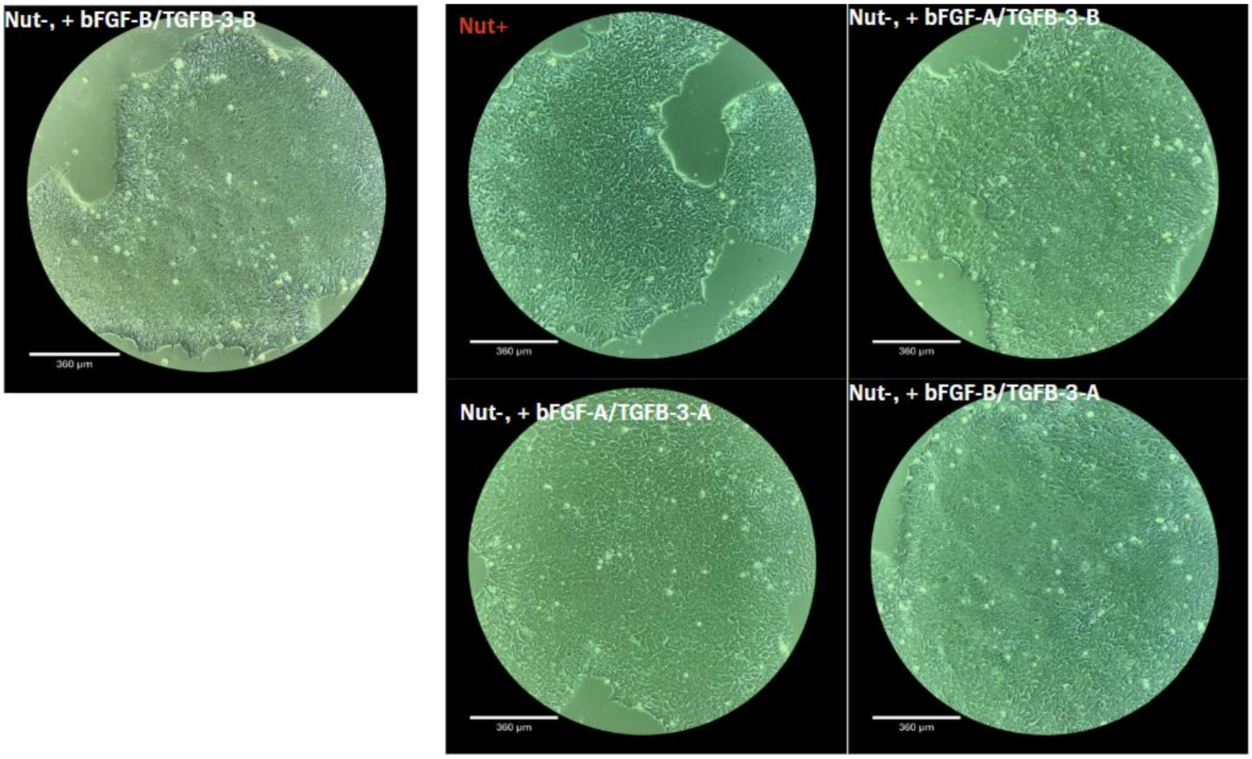

Figure S6. Morphology assessment comparing different combinations of bFGF and TGF-β3 from Vendors A and B. Representative brightfield images of hPSC colonies on Day 6 at 4× magnification.

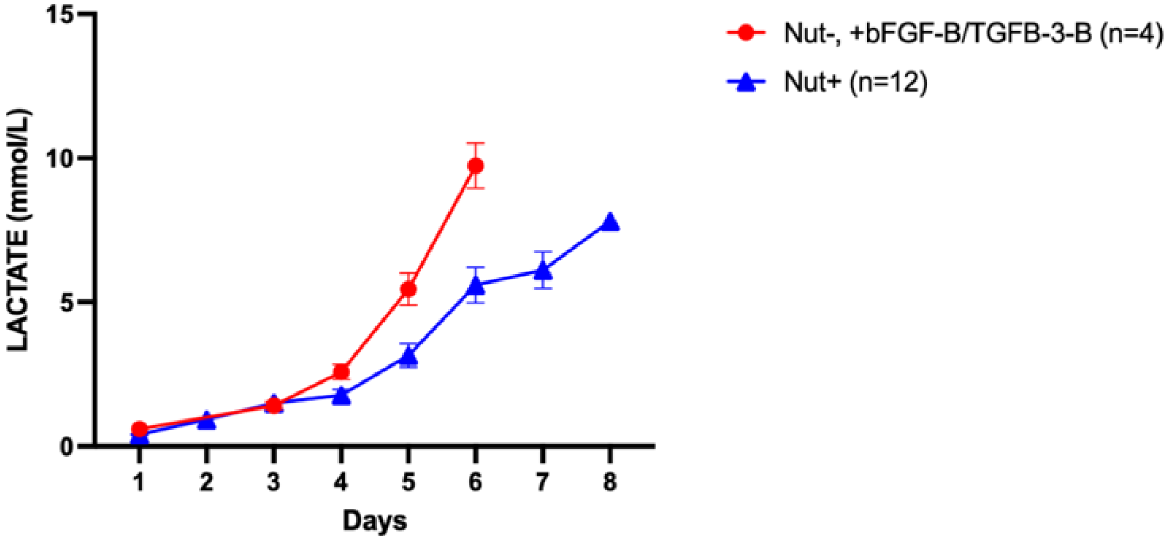

Figure S7. Lactate measurements performed in large-scale hESC cultures (75-cm² T-flasks) for Nut+ (control) and across four independent lots of Nut− base medium supplemented with bFGF-B/TGFB-3-B.

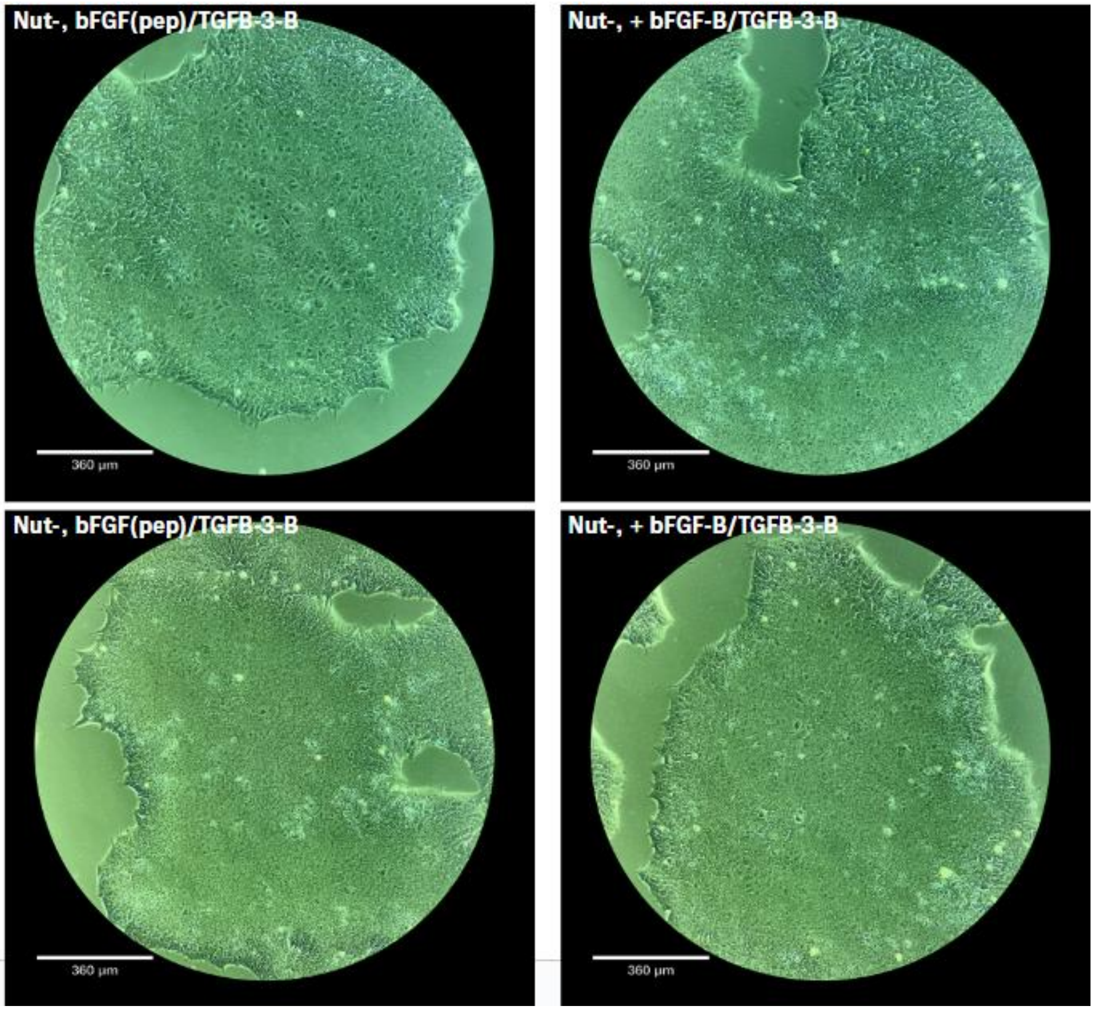

Figure S8. Morphology assessment comparing the peptide FGFR agonist (bFGF(pep)) and bFGF-B. Representative brightfield images of hPSC colonies on Day 6 at 4× magnification.
